# Telerehabilitation Solution Conceptual Paper for Community-Based Exercise Rehabilitation of Patients Discharged After Critical Illness

**DOI:** 10.5195/ijt.2016.6205

**Published:** 2016-12-15

**Authors:** APHRODITE TSAVOURELOU, NIKOLAS STYLIANIDES, ANDREAS PAPADOPOULOS, MARIOS D. DIKAIAKOS, SERAFEIM NANAS, THEODOROS KYPRIANOY, SAVVAS P. TOKMAKIDIS

**Affiliations:** 1 CRITICAL CARE DEPARTMENT, NICOSIA GENERAL HOSPITAL, NICOSIA, CYPRUS; 2 EUROPEAN UNIVERSITY CYPRUS, SCHOOL OF SCIENCES, DEPARTMENT OF HEALTH SCIENCES, NICOSIA, CYPRUS; 3 OPEN UNIVERSITY OF CYPRUS, APPLIED HEALTH INFORMATICS, NICOSIA, CYPRUS; 4 DEPARTMENT OF COMPUTER SCIENCE, UNIVERSITY OF CYPRUS, NICOSIA, CYPRUS; 5 FIRST DEPARTMENT OF CRITICAL CARE, SCHOOL OF HEALTH SCIENCE, NATIONAL AND KAPODISTRIAN UNIVERSITY OF ATHENS, “EVANGELISMOS” HOSPITAL, ATHENS, GREECE; 6 ST GEORGES UNIVERSITY OF LONDON MEDICAL PROGRAM MBBS4, UNIVERSITY OF NICOSIA MEDICAL SCHOOL, NICOSIA, CYPRUS; 7 SCHOOL OF PHYSICAL EDUCATION AND SPORT SCIENCE, DEMOCRITUS UNIVERSITY OF THRACE, KOMOTINI, GREECE

**Keywords:** Cost benefit analysis, Intensive care, Rehabilitation, Tele-monitoring, Telesupervised exercise

## Abstract

A novel service oriented platform has been developed under the framework of the Telerehabilitation Service funded by the Cross Border Cooperation Programme Greece Cyprus 2007 – 2013 to support tele-supervised exercise rehabilitation for patients after hospitalization in intensive care units (ICU). The platform enables multiparty, interregional bidirectional audio/visual communication between clinical practitioners and post-ICU patients. It also enables patient group-based vital sign real time monitoring, patients’ clinical record bookkeeping, and individualized and group-based patient online exercise programs. The exercise programs intended for the service are based on successful cardiorespiratory rehabilitation programs, individualized and monitored by a multidisciplinary team. The eligibility study of former ICU patients to participate in such a service as well as a cost benefit analysis are presented to support the cost effectiveness of the telerehabilitation program in addition to the expected health benefits to a large proportion of former ICU patients.

Most of the patients who are hospitalized in intensive care units (ICUs) recover (Elias et al., 2015) and return home (Flaatten, 2010, Meynaar et al., 2012, NICE, 2009). However, post-ICU patients suffer not only from diagnosis-related organ dysfunction of variable severity, but also from neuromuscular weakness, reduced functional capacity, exercise tolerance, health related quality of life (HRQoL) and social function (Flaatten, 2010, Herridge et al., 2016). Reduced exercise tolerance and functional capacity in these patients, is not solely attributed to their acute or chronic organ dysfunctions, but also to the so-called ICU acquired weakness, a multi-factorial syndrome related to inflammatory injury of nerve and/or muscle which is exacerbated by sarcopenia, functional denervation and corticosteroids (Griffiths & Hall, 2010).

In most cases, there is no specialized support to cope with the aforementioned long-term problems of the patients discharged from ICUs (Connolly et al., 2014). At present, programs after a period of stay in the ICU are specific for each disease and are offered in some cases from neurosurgery, cardiology and burns clinics. For patients who do not fall into these disease-specific categories, there is no alternative path of rehabilitation.

Systematic exercise in the context of diagnosis-specific rehabilitation programs can positively influence the course of physical and non-physical problems associated with acute and/or chronic pulmonary (Ries et al., 1995) or cardiovascular diseases (Izawa et al., 2010). In cardio-respiratory rehabilitation programs, the improvement of physical condition and function is achieved by specific exercises of the upper and lower limbs. Aerobic exercise and muscle strengthening, when applied systematically and with the appropriate individualized methodology (Garber et al., 2011) helps patients with decline in cardio-respiratory and neuromuscular function to restore their autonomy, improve their psychological status and eventually their quality of life (Morey et al., 2009, NICE, 2009, Rees et al., 2004). It is likely that such an approach could help ICU patients to return to their baseline (before ICU admission) level of activity and may improve their health and physical condition even more. In addition, it could reduce health care related burden and costs at both primary and secondary levels (medication use, GP and specialty visits, hospital readmissions, complications etc.). Therefore, it is essential that safe and effective rehabilitation programs for health improvement in post-ICU patients be available on either a hospital or community basis.

An effective integrated rehabilitation program should consist of exercise training, educational, psychological and nutritional support, with exercise being the core component (Connolly et al., 2012). Recent pilot studies, provide preliminary data for potential, exercise-oriented rehabilitation strategies post ICU (Batterham et al., 2014, Berney et al., 2012, Connolly et al., 2012, Connolly et al., 2015, Denehy et al., 2013, Elliott et al., 2011, Jones et al., 2003, Lau et al., 2005, McWilliams et al., 2009, McWilliams et al., 2016, Salisbury et al., 2010). Some of these studies present positive results regarding the efficacy of such interventions (Jones et al., 2003, Lau et al., 2005, McWilliams et al., 2009, McWilliams et al., 2016), whereas other studies might be used as a starting point for future research.

Despite the positive effects of exercise rehabilitation programs, it is not often feasible for patients to join such programs. The exercise programs are not usually accessible to patients either because their situation does not allow easy transportation, or because they imply serious economic expenses and time consuming transportation. Due to the lack of competent exercise programs, patients often fail to regain their skills and their status can gradually deteriorate with impact on mortality, morbidity, and extra cost for their families and health system (Loyalka et al., 2014, Snook & Webster, 1987). Exercise telerehabilitation programs could surpass the above obstacles, increase accessibility and enhance adherence for post ICU patients as well as for other vulnerable populations such as people with disabilities (Demiris et al., 2005, Winters, 2002). Furthermore, a number of studies suggest that exercise rehabilitation programs may be provided remotely, leveraging information technology and telecommunications as means delivery (Shukla et al., 2016, Sugarman et al., 2006, Tousignant et al., 2006, Winters, 2002, Zanaboni et al., 2016, Zanaboni et al., 2016).

As reported in a systematic review by Hailey et al. (2011) the success of tele-supervised exercise programs (telerehabilitation) for specific groups of patients has been demonstrated by several studies. Indeed, the systematic review of 11 telerehabilitation trials for the secondary prevention of coronary heart disease (Neubeck et al., 2009) showed significant improvements on CHD risk factors including exercise adherence and volume, and positive alterations in total cholesterol, high density lipoprotein cholesterol and systolic blood pressure. While studies conducted with people in the chronic phase following stroke, as described in the review of Laver et al. (2013), might have provided insufficient evidence of effectiveness, they indicated that remotely assisted exercise programs in this population are feasible. In 13 of the 19 studies reviewed by Hailey et al. (2013) the application of telerehabilitation in patients with neurological conditions provided at least equivalent outcomes to conventional approaches. Furthermore, both health professionals and patients reported high satisfaction and acceptance of telerehabilitation interventions (Johansson & Wild, 2011).

## NICOSIA GENERAL HOSPITAL TELEREHABILITATION SERVICE

A telerehabilitation platform^1^ is being actively used by the Nicosia General Hospital. The platform has been developed under the framework of the Telerehabilitation Service funded by the Cross Border Cooperation Programme Greece Cyprus 2007–2013. It provides remote services for home care and rehabilitation for patients in the community. The professional staff is responsible for managing, monitoring, and evaluating the rehabilitation groups. Table 1 depicts the role-based responsibilities of the staff. Patients who are selected to participate in the program, follow a 12-week exercise rehabilitation program (Table 2). An exercise program adapted to the patient’s needs is determined by an ICU physician, an ergophysiologist and a physiotherapist after initial assessment. For patients with heart failure or respiratory failure, the exercise program is determined by an external expert.

**Figure 1:**
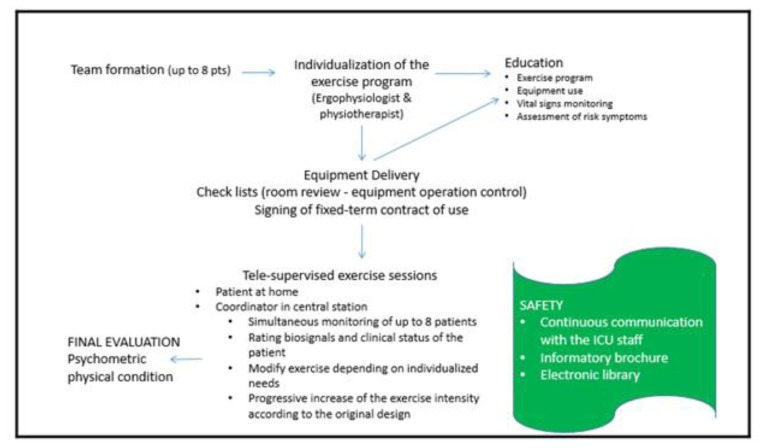
Diagram of the Nicosia General Hospital telerehabilitation service.

Patients, selected to participate in the program, need to follow a 12-week exercise training rehabilitation program. The exercise program constitutes of consecutively continuous and interval aerobic exercise, endurance exercise and stretching (Table 2). Endurance exercises progress every week (Table 2) following assessment of patients’ biosignal responses to current set of exercises, dyspnea, psychological factors etc. After initial assessment of the patient, the training exercise program adapted to the patient’s needs is determined by an ICU physician, a clinical exercise physiologist, and a physiotherapist. For patients with heart failure or respiratory failure, the exercise program is determined by the clinical exercise physiologist in collaboration with the patient’s cardiologist. A comprehensive diagram of the Nicosia General Hospital Telerehabilitation service is presented in Figure 1.

### SOLUTION DESCRIPTION

The telerehabilitation platform being used by the Nicosia General Hospital consists of mature, fine-grained services and supports real-time/online and offline interactions between health professionals and patients (Stylianides et al., 2013). An exercise rehabilitation session takes place in real-time in a Virtual-Room between a group of no more than eight patients under the coordination of a health professional. The platform supports real-time bidirectional transfer of high definition video (image and sound) between the coordinator (central station) and the participating patients (home stations). In addition to the high definition video, the platform records all of the patients’ biosignals, i.e. 3-lead ECG, Heart Rate, Oxygen Saturation and Blood Pressure, in real time. This information (video, audio, biosignals) is presented through a user-friendly interface. An automated procedure allows the patients to seamlessly join the virtual rooms and attend the exercise sessions.

Furthermore, the offline interaction of the patients with the platform allows the browsing of the exercise rehabilitation sessions mainly for educational purposes. Additionally, qualified members of the telerehabilitation staff (ergophysiologists/physiotherapists), can manage offline (create, update, and delete) rehabilitation groups and teleconference sessions (i.e., to assign patients, caregivers, health professionals, educators, trainers, and coordinators for the sessions). Finally, the platform includes Folder Automation Software of patients’ rehabilitation methodology that securely stores the patients’ records.

Telerehabilitation sessions are monitored and coordinated through specially designed and equipped central stations. Specifically, central stations are equipped with broadband internet access, videoconferencing system, monitoring system of the patients’ biosignals, sound system (microphones, loudspeakers and amplifier), and video recording system, in order to remotely monitor the image and the biosignals of patients.

A central station can control simultaneously up to eight home stations. A home station is provided to each patient and supports teleconference in virtual rooms, biosignals monitoring and transmission to central stations, online training and consulting and direct communication (even out of the program preset rehabilitation sessions) with remote rehabilitation center, using a network broadband infrastructure (at least 768Kbps uplink, static internet address, correlation with predetermined domain name, domain name service). To enable biosignals monitoring and transmission, wearable lightweight, portable (battery powered) wireless (802.11b) devices with biosignal sensors are involved. Specifically, the wearable devices monitor and transmit (via direct connection) to a central unit the patient’s biosignals; that is, 3-lead electrocardiogram, derived respiration rate (Moody et al., 1985), blood pressure (non-invasive), oxygen saturation (pulse oximetry) and accurately estimated heart rate. A belt, attached to the patient’s waist and shoulder, guarantees security and stability of the devices. Furthermore, to facilitate the exercise rehabilitation program, the following exercise equipment accompanies a home station: (a) electromagnetic legs ergometer that features adjustable workload from 10 to 350 watt (interval steps of 5 watt) directly from the console, a flywheel ≥ 6Kg, vertical and horizontal adjustable handlebars, vertical and horizontal user adjustable saddle, wide gel padded seat and pedals with quick adjustment strap. A leg ergometer is adjusted to each patient during the first assessment; (b) resistance bands from 2lbs to 13lbs (6 color-coded resistance levels). Each patient gets several bands of different color (for easy identification) and resistance level as required by the patient’s resistance bands exercise set. The resistance level for each exercise is decided after assessment at the beginning and at the middle of the tele-supervised exercise program; and (c) soft weights of 0.5kg to 3kg (6 weight levels) which are also individually assigned to patients according to the initial and mid assessment of the resistance exercises.

### PLATFORM SOFTWARE AND HARDWARE COMPONENTS

The telerehabilitation platform consists of the following software/hardware components:

#### VIDEO COMMUNICATION SYSTEM

Each central station is accompanied by dedicated video conferencing equipment. Particularly, video and audio communication is established using business solution VoIP video conference (VC) systems. The Video Conference System leverages a MCU (Multiparty Control Unit) that enables the creation of virtual rooms, automatically establishes calls with all participants (telerehabilitation patients and clinicians), configures the clinician’s view in tile window layout and starts session recording based on pre-scheduled scenarios. The recording of sessions is performed by the Video Center service for further evaluation. To support this, home stations come with specialized video-conferencing software and are equipped with USB high definition (HD) cameras and microphones.

#### CLINICAL INFORMATION SYSTEM

Patient clinical data are monitored during the telerehabilitation session using in-hospital clinical information system solutions and the wearable devices that accompany home stations. Specifically, all participating patients are being concurrently monitored and their vital signs are transferred through a central unit to the conference system, thus enabling interregional monitoring and data storage. All data are transferred over virtual private network (VPN - IPSec no-compression) tunnels utilizing less than 100Kbps. Moreover, vital signs are exported offline to the Clinical Report Form (CRF) System in HL7 format.

#### WEB BASED SERVICES

The Patient Management System (PMS) effectively and securely manages patient medical data. PMS consists of the following systems: *(a) Patient Group Scheduling and Management System* that allows qualified health professional to manage (create, update, and delete) rehabilitation groups and teleconference sessions; *(b) Online Training System* that facilitates tele-supervised individualized or group based training sessions. A patient’s station is programmed with a predefined time interval for its training material (single page textual or/and visual information) or consultation. The clinician in charge is able to assign the appropriate training material to a group of patients and/or to each patient individually during a session or offline; and (c) *Clinical Report Form System (CRF)* that manages private and non-private patient medical data. It consists of two individual services each handling only one class of medical data (private and non-private). CRF stores more than 100 individual fields and eight health surveys and medical examinations. Non-private data are exported to comma separated value (csv) files for further processing using statistical analysis software such as SPSS. All these systems follow the model-view-controller (MVC) software architecture, are written in php language, run on apache web server and leverage the MySQL relational database management system for medical records storage.

### CLINICAL RECORD SECURITY

To protect clinical records, the private and non-private data are stored and processed in separate servers. Private data are any data that could, directly or indirectly, identify a person. That is, an identification number (i.e., identity card number, passport number, etc.) or anything that specifies the patient’s physical, physiological, mental, economic, cultural or social view (i.e., demographic data). Private data are encrypted, using AES symmetric-key algorithm; prior storage and access is allowed only from the server itself. Access to the private-data server is restricted to health professionals only. Moreover, the roles of health professionals (i.e., doctor, secretariat, physiotherapist, nurse, clinical exercise psychologist, research coordinator, IT administrator and homecare support group) are of major importance. Particularly, health professionals have access to specific data views for specific patients based on their role (i.e., the psychologists cannot view demographic data and ICU scores). Only the research coordinator has full access to the data. Lastly, all servers use encrypted file systems to avoid direct access to the data or the source code, and backups are created and securely stored on a regular basis.

### CHALLENGES

The synergy of heterogeneous technology systems in the same infrastructure allowed the effective implementation of the described telerehabilitation platform. Among the technology systems used were teleconferences, monitoring of biosignals, electronic patient records and projection systems of customized training materials. Moreover, the huge number of real time streaming patients’ biosignals should be transmitted, displayed, and stored efficiently and effectively in real time. In addition, the adaptation of technology to specific characteristics and skills of different service users (e.g., health professionals, technical staff and relatives of patients) as well as the reaction of users to new methodologies, technologies and practices, presented a lot of difficulties. Finally, the education of health professionals in a diverse (regarding issues of services and systems) technological infrastructure was not an easy task. It was required to prepare manuals and guidelines of use and management of the infrastructure services as well as clinical practice guidelines.

## PATIENT ELIGIBILITY AND FINANCIAL APPRAISAL

Before commencing the implementation of such a challenging service it was necessary to assess the eligibility of former ICU patients to participate, and for researchers to perform a cost benefit analysis.

### ELIGIBILITY STUDY VERIFICATION

This pilot verification study was undertaken in order to assess eligibility for inclusion of former ICU patients in a tele-supervised exercise training program, applying certain exclusion criteria (pertaining to low probability of ICU related disability and to physical disability). In addition, the study aimed to define the prevalence of these limiting factors.

Thus, a prospective study of consecutive admissions to the general ICU of Nicosia General Hospital for the eligibility to participate in a home-based telerehabilitation program was conducted (multi party video conference and vital sign monitoring sessions). The *exclusion criteria* for participation were: (a) mechanical ventilation for less than 48 hours; (b) poor neurological outcome as expressed by a Mini Mental Scale Examination score <23/30; (c) poor functional ability as expressed by a Rivermead Mobility Index <8/15; (d) end stage chronic illness; and (e) hospital discharge to chronic health care facilities or other hospitals.

The data were collected from February 2013 until October 2013. The analysis was conducted on 451 patients; 77 (17%) died (hospital mortality), 55 (12%) were considered eligible and 319 (70%) were non-eligible.

Three hundred nineteen non-eligible patients were excluded because of (a) Mechanical Ventilation < 48 hrs 240 (75%), (b) poor neurological outcome (Mini Mental Scale Examination <23/30) 39 (12%), (c) poor functional ability (Rivermead Mobility Index < 8/15) 7 (2%), (d) end stage chronic illness 12 (4%), (e) hospital discharge to chronic health care facilities or other hospitals 21 (7%) (Table 3). Percentages are from the total of non-eligible patients (319=100%).

### FINANCIAL APPRAISAL

The cost benefit analysis of the telerehabilitation program was based on a discounted cash flow (DCF) analysis, which estimates the return of investment adjusted for the time value of money using as measures of success the net present value (NPV) at a declared cost of money percentage and the internal rate of return (IRR), both for a declared financial period. DCF analysis is generally considered the best way to assess investment projects. The major advantage is that it takes into account the fact that a certain amount of money today is worth more than the same amount in the future, mainly because of inflation. Such an analysis indicates that a project is likely to be “profitable” – increase the investors’ wealth, when (a) the Net Present Value of the project (NPV) is positive and when (b) the project’s rate of return (IRR) is higher than the cost of capital (rate of return is the rate which results to zero net present value of the project) (Sartori et al., 2015).

The financial projections assumed an increasing number of Telerehabilitation users (both rehabilitation sessions and successive follow-ups) over a 5-year period. Relevant investigation, as mentioned above, showed that approximately 75–96 patients would use the service each year. Thus, in the fifth year, 122 full users and 200 follow-up patients would use the Telerehabilitation facilities. The initial investment for the entire technical equipment (including devices, systems, software etc.) is estimated to be 382,000 Euros. Consequently, the analysis established that both measures were positive (Table 4). The sum of cash flows (costs and benefits included) of every year in a five-year period was calculated with an annual discount rate of 10%, resulting in 30,000 Euros NPV for Tele-Rehabilitation. The IRR was calculated to be 11.8% which is higher and thus, better compared to a standard discount rate of assumed 10% (Table 4).

## CONCLUSION

The prospective study of consecutive admissions to the general ICU of Nicosia General Hospital gives preliminary evidence that a large proportion of ICU patients are eligible to participate in a planned home-based exercise rehabilitation program. Specifically, 12% of all admissions to the ICU of Nicosia General Hospital during an 8-month period were eligible to participate. Physical disability deterring participation was observed in 14% (12 with mental deficit plus 2 with mobility deficit). The majority of the non-eligible patients (75%) were patients with ICU stay less than 48hrs; therefore, patients with low probability of ICU related disability. A telerehabilitation program for former ICU patients could be addressed with some changes from the above described, not only for the eligible patients of this study but also for patients with poor neurological outcome, poor functional ability, end stage chronic illness and probably hospital discharge to chronic health care facilities or other hospitals. These factors represent approximately 30% of the total patients. This suggests that one-third of former ICU patients can participate in a home-based telerehabilitation program. Furthermore, the above mentioned one-third includes the vast amount of patients who need rehabilitation after ICU hospitalization.

Likewise, as shown by the financial appraisal of the service, the telerehabilitation program is worth the initial investment and leads to better financial output compared to re-hospitalization of patients because of incomplete rehabilitation/recovery. This is implied by the fact that the calculated cost of a former ICU patient telerehabilitation program is about the same with one day of readmission in ICU (Dasta et al., 2005, Judson & Fisher, 2006). Furthermore, NPV and IRR gave a calculation of predicted benefit for the first five years of operation of the designed telerehabilitation service which supports that such an investment is profitable.

In summary, the design and implementation of a telerehabilitation platform to meet the needs of former ICU patients was a demanding task. The financial appraisal, in addition to the expected health benefits to a large proportion of former ICU patients renders such a project necessary in our society.

## Figures and Tables

**Table 1:** Roles and Tasks of the Telerehabilitation Staff

Role	Tasks
Doctor	- Maintain records of patient history - Perform clinical evaluation before, during, and after a patient’s rehabilitation program Optimize medication - Assess the patient’s exercise capacity - Individualize the rehabilitation program and optimal load for each patient - Educate patients on self-assessing worrying signs and their level of dyspnea during exercise - Communicate with patients via telephone (if necessary as assessed by the nursing staff) - Manage emergencies - Obtain (signed) informed patient consent to participate in the rehabilitation program
Nurse	- Maintain records of patient history - Provide patient education - Communicate with patients via telephone about potential problems and their need of medical advice - Manage emergencies - Ensure that patients complete all necessary forms during participation in the program - Record, distribute, and ensure the functionality of the technological and fitness equipment - Perform anthropometric measurements before and after the rehabilitation program
Ergophysiologist	- Assess each patient’s physical ability - Design an exercise program and the optimal load for each patient - Educate patients on how to perform the exercises prescribed and assess the adequacy of training
Physiotherapist	- Coordinate the exercise program - Maintain clinical record bookkeeping - Select exercises for each patient and post them on their computer - Include patients in each session - Analyze the electrocardiogram (ECG) in consultation with the doctor - Evaluate the patient’s level of dyspnea and vital signs during exercise - Assess each session of the exercise program and modify ineffective exercises - Provide advice via telephone communication - Maintain records of patient history
Psychologist	- Provide psychological evaluation - Provide psychological support

**Table 2:** Exercise Rehabilitation Program; General Components

8 weeks-3 times/week - 24 sessions		
Aerobics	30 min	Alternating: Continuous [30min in 70% VO2p]/interval [30″ to 100 VO2p-30″ passive break]
Resistance training	20 min	6 exercises (upper and lower extremities-trunk) Up to 4 set × 10–15 reps (progressively)
Recovery	10min	Stretching

**Table 3:** Non-eligibility Frequency Table

		Frequency	Percent (%)	Valid Percent (%)	Cumulative Percent (%)

Non-eligible	MV<48	240	53.4	75.3	75.3
	MMSE<23	39	8.6	12.2	87.5
	RMI<8/15	7	1.6	2.2	89.7
	End stage chronic illness	12	2.7	3.8	93.4
	Transfer to other facility	21	4.7	6.6	100.0
	Total	319	71.0	100.0	
Missing	System	132	29.0		
Total patients		451	100.0		

MV: Mechanical ventilation, MMSE: Mini Mental State Examination, RMI: Rivermead Mobility Index

**Table 4:** Discounted Cash Flow Analysis

Operating time	before-operation	1	2	3	4	5
	€	€	€	€	€	€
Initial company value- before investment	−382,000	−10,000	−10,000	−10,000	−10,000	−10,000
Operating liquidity		15,400	48,640	19,040	46,580	112,620
loan repayment			0	0	0	0
residual value						450,192
	−382,000	5,400	38,640	9,040	36,580	552,812
Net Present Value (NPV)	10.00%	€ 29,872				
Internal Rate of Return (IRR)		11.8%				

## Footnotes

1 http://telerehabilitation.cs.ucy.ac.cy/en
