# Correction To: TSAVOURELOU, A., STYLIANIDES, N., PAPADOPOULOS, A., DIKAIAKOS, M. D., NANAS, S., KYPRIANOU, T., TOKMAKIDIS, S. P. (2016). TELEREHABILITATION SOLUTION CONCEPTUAL PAPER FOR COMMUNITY-BASED EXERCISE REHABILITATION OF PATIENTS DISCHARGED AFTER CRITICAL ILLNESS. INTERNATIONAL JOURNAL OF TELEREHABILITATION, 8(2), 61–70. DOI: 10.5195/IJT.2016.6205

**DOI:** 10.5195/ijt.2017.6223

**Published:** 2017-06-29

**Authors:** APHRODITE TSAVOURELOU

The sixth author’s name was misspelled. It should read: Kyprianou. A corrected version has been made available at https://doi.org/10.5195/ijt.2016.6205.

